# Eat, Train, Sleep—Retreat? Hormonal Interactions of Intermittent Fasting, Exercise and Circadian Rhythm

**DOI:** 10.3390/biom11040516

**Published:** 2021-03-30

**Authors:** Sandra Haupt, Max L. Eckstein, Alina Wolf, Rebecca T. Zimmer, Nadine B. Wachsmuth, Othmar Moser

**Affiliations:** 1 Division of Exercise Physiology and Metabolism, Department of Sport Science, University of Bayreuth, 95440 Bayreuth, Germany; sandra.haupt@uni-bayreuth.de (S.H.); max.eckstein@uni-bayreuth.de (M.L.E.); alina.wolf@hs-coburg.de (A.W.); rebecca.zimmer@uni-bayreuth.de (R.T.Z.); nadine.wachsmuth@uni-bayreuth.de (N.B.W.); 2 Department of Social Work and Health, Coburg University of Applied Sciences, 96450 Coburg, Germany; 3 Interdisciplinary Metabolic Medicine Research Group, Division of Endocrinology and Diabetology, Medical University of Graz, 8036 Graz, Austria

**Keywords:** intermittent fasting, circadian rhythm, exercise, metabolism, stress hormones

## Abstract

The circadian rhythmicity of endogenous metabolic and hormonal processes is controlled by a complex system of central and peripheral pacemakers, influenced by exogenous factors like light/dark-cycles, nutrition and exercise timing. There is evidence that alterations in this system may be involved in the pathogenesis of metabolic diseases. It has been shown that disruptions to normal diurnal rhythms lead to drastic changes in circadian processes, as often seen in modern society due to excessive exposure to unnatural light sources. Out of that, research has focused on time-restricted feeding and exercise, as both seem to be able to reset disruptions in circadian pacemakers. Based on these results and personal physical goals, optimal time periods for food intake and exercise have been identified. This review shows that appropriate nutrition and exercise timing are powerful tools to support, rather than not disturb, the circadian rhythm and potentially contribute to the prevention of metabolic diseases. Nevertheless, both lifestyle interventions are unable to address the real issue: the misalignment of our biological with our social time.

## 1. Introduction

Humans are responsive to a multitude of metabolic and hormonal “background” processes dictating daily life [1]. From the days of the Stone Age until the 21st century, humans have submitted to the fundamental daily light/dark cycle on Earth, which is the foundation of our wake/sleep cycle called the circadian rhythm [2]. This highly complex cycle has a major influence on our metabolic and hormonal health [3,4]. Throughout human evolution, wake/sleep cycles have influenced our lives as much as our dietary intake. The human genome evolved during the hunter-gatherer period, which led to a selection of genes and traits that define the humane gene pool today [5,6]. A major contributing factor in defining our genome is the interplay of food scarcity and food abundance that accompanied humans throughout their evolution, enabling human metabolism to store surplus energy [7,8].

In earlier days, extended periods of fasting led to metabolic stress, which increased the demand for food or promoted pathways of energy preservation by extending sleep periods. Across all species on earth, sleeping behavior has shown a remarkable flexibility to counteract metabolic stress, whereas the circadian rhythm remains the fundament, yet duration, sleep architecture and timing of sleep may adapt autonomously.

With industrialization and the ubiquitous availability of food, the ability of the human body to be as efficient as possible at storing surplus energy gave rise to several common diseases and the “global burden of diabetes” [9]. However, this burden to public health has not only emerged due to a disturbed metabolic milieu leading to an excessive gain in body weight but also due to our modern lifestyle, which is characterized by physical inactivity and overconsumption of food [10,11]. Our metabolism demands physical activity since similar to our metabolic response to the absence/presence of food, our genomes support a “physical activity cycle” as discussed by Chakravarthy et al., which, if ignored can be an origin of disease [6]. The sheer complexity in maintaining a balanced calorie intake, sufficient physical activity and adequate sleep has previously been the subject of investigation [12,13]. However, the hormonal background, potential interactions and confounding variables remain a matter of investigation.

In recent years, intermittent fasting in particular time restricted feeding, described as the limited consumption of foods and calorie-containing beverages in a set window of a few hours per day and constantly abstaining from calorie-dense products for the remaining hours, has shown health benefits [14,15]. The effects of fasting and the interaction with the circadian rhythm and its hormonal response in “re-setting the clock” have previously been discussed in several reviews [16,17,18]. However, it is unclear how physical exercise can be incorporated into this complex metabolic and hormonal interaction. Even though advocated as the “poly-pill” for the treatment of certain diseases, prescribing exercise in type, intensity, duration and mode may have a major impact on circadian rhythm and intermittent fasting strategies [19,20]. Therefore, this present review aimed to summarize novel information on fasting strategies, intermittent fasting, circadian rhythm and exercise with respect to metabolic and hormonal responses to define recommendations of how to incorporate exercise for a healthy lifestyle.

## 2. Materials and Methods

For this narrative review, a literature search on PubMed was conducted in December 2020 for research studies involving intermittent fasting (16/8), circadian rhythm, metabolic pathways and their interaction with physical exercise. Key articles from these broad areas of research were included.

## 3. Results

### 3.1. Circadian Rhythm

The obligate need for a chocolate brownie after lunch and craving for sweet beverages during the day when actually being overfed, or being overexcited when you are tired, sometimes seems like an arbitrary reaction of the body. Nevertheless, metabolic processes in the human sleep/wake and feeding/fasting cycle are influenced by a complex network of circadian pacemakers. They are synchronized by the suprachiasmatic nucleus (SCN) [21,22], which is located at the base of the hypothalamus, functioning as a central clock, and several peripheral tissue-specific clocks. The rhythmicity, which is sculpted by a central transcription-translation autoregulatory feedback loop [23], roughly reflects the 24 h daily rhythm [24]. Anticipatory adjustments of rhythmicity of both, the SCN and peripheral clocks, are induced by changes in the exogenous physical environment. This leads to the formation of individual circadian rhythms. These rhythms interact with several physiological processes, like sleep homeostasis [25,26,27], metabolic function [28,29,30] and the immune system [31,32]. Additionally, numerous peripheral modulators are responsible for the control of tissue-specific processes. They are partly located in other brain regions like the pineal or pituitary gland [33], but are also localized outside the brain like cardiomyocytes, liver and muscle cells [34,35,36,37].

#### 3.1.1. Hormonal Pathways and Its Effects on the Circadian Rhythm

The circadian rhythmicity in mammals is driven by the primary transcriptional-translational autoregulatory feedback loop in the SCN including the key transcriptional activators, circadian locomotor output cycles kaput (CLOCK) and brain and muscle Arnt-like 1 (BMAL1) [38]. The CLOCK:BMAL1 heterodimer subsequently formed initiates the expression of Period (*Per*) and Cryptochrome (*Cry*) genes. Specific messenger ribonucleic acid (mRNA) is then translated into the mPER and mCRY proteins in the cytoplasm. The PER:CRY heterodimers translocate to the nucleus and inhibit their own transcription by interacting with CLOCK:BMAL1 [24,39,40,41]. mPER and mCRY proteins form a part of the negative limb of the feedback loop. A second regulatory loop is initiated by CLOCK:BMAL1-activated transcription of retinoic acid-related orphan receptors *Rev-erbα,β* and *Rorα,β,γ*. The translated proteins, REV-ERBα,β and RORα,β,γ, bind to retinoic acid-related orphan receptor response elements (ROREs). ROREs, which are present in the BMAL1 promoter, modulate transcription of Bmal1. REV-ERBα,β and RORα,β,γ influence expression in different ways. RORα,β,γ enhances *Bmal1* mRNA levels, while REV-ERBα,β represses them [42,43]. Both proteins are crucial in modulating the positive site of the autoregulatory feedback loop. Besides the central clock gene (CG) expression, many other peripheral cells show a rhythmical oscillation of CGs, as seen in liver, gut, heart, testis or adipose tissue [44,45,46,47]. In addition to CGs, peripheral tissues show transcription of thousands of clock-controlled genes (CCGs) regulated by clock output genes [48]. They influence, for example, the circadian rhythm of endocrine processes, including the formation of adrenocorticotropic hormone (ACTH) or other glucocorticoids [49,50] or rhythmicity of metabolism [51,52].

#### 3.1.2. External Stressors of the Circadian Rhythm

**Figure 1:**
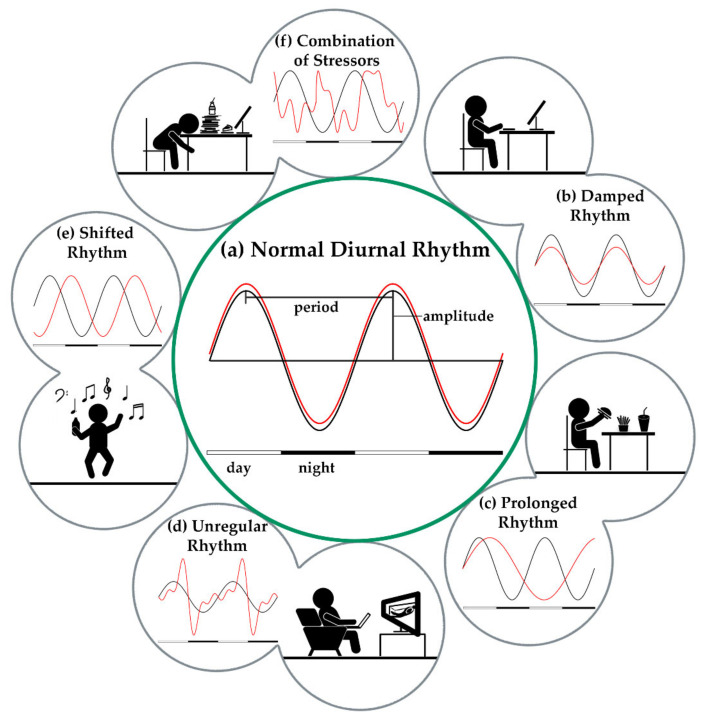
Different Stressors modify the normal diurnal rhythmicity. The circadian rhythm is controlled by the light/dark-cycle that influences the rhythmicity of central pacemaker (black) and thus all peripheral processes (red) are synchronized with each other (**a**). Different external stressors can lead to disturbances in the highly complex interaction of the central and peripheral oscillators. Damped responses of peripheral pacemakers (**b**) are observed especially in insulin resistance or night eating. High fat meals lead to prolonged cycles (**c**) and thus a shift of the periods against each other. Irregular rhythms (**d**) may be caused by irregular meals or generally an irregular lifestyle. A decoupling of the sleep/wake cycle from the light/dark cycle, as seen in shift work or due to modern lifestyle prolonged waking times, can additionally shift the periods against each other (**e**). A combination of several factors can result in a partial or complete decoupling of the peripheral from the central pacemaker (**f**).

Whenever the natural circadian rhythm is altered by external factors, this has physiological (metabolic stress) as well as psychological (cognitive function and mental health) consequences [53]. The level of information about potential relationships between shift work and various diseases of civilization such as various types of cancer, metabolic or cardiovascular disease, or in combination, can be considered as inconsistent and rather inconclusive [54]. However, a factor for an increased risk of developing disease is a shifted rhythm of the sleep hormone melatonin, which has been shown previously [55,56]. Melatonin is responsible for controlling thermoregulatory processes. The lowering of core body temperature when melatonin is released, results in the onset of sleepiness in the evening and a drop in melatonin concentration in the morning leads to awakening [57]. Exposure to light at night leads to an inhibition of melatonin release and, in shift workers, results in a shortened over-all sleep duration on workdays [58] and an increased prevalence of sleep disorders due to a shifted sleep-wake cycle [59,60]. In the morning, decreasing melatonin levels go hand in hand with formation of cortisol, which is regulated by the hypothalamic-pituitary-adrenal (HPA) axis. Elevated total cortisol levels right after waking up [61] as well as a rise in absolute cortisol concentrations [62] were observed in shift workers previously. This indicates a disruption of the pituitary-adrenal response to light stimuli, which can already be observed after only five days of nighttime work [63]. The influence of increased cortisol and thus decreased melatonin levels on glucose metabolism was reviewed by Cipolla-Neto et al. They discussed studies on pinealectomized animals with a loss in endogenous pineal melatonin production, showing an increased peripheral and central insulin resistance, as well as decreased glucose tolerance and a reduction in glucose transporter type 4 (GLUT4) mRNA in adipose and muscle tissue [64,65,66]. Therefore, an appropriate melatonin replacement therapy for populations with a lack in diurnal melatonin production, like in shift workers and in the elderly, for either preventing, eliminating, or both, metabolic diseases is recommended [67]. This demonstrates the distinct influence of shift work on glucose homeostasis. Shift work also affects other endocrine functions such as the secretion of ghrelin and leptin. Leptin, a satiety hormone produced by the adipose tissue, is important in regulating appetite and energy metabolism [68]; its synthesis is regulated by nutritional status. Ghrelin can be understood as an antagonist to leptin and is produced in the pancreas and the gastric mucosa. Higher ghrelin levels promote a decreased fat oxidation that leads to an increase of adipose tissue and food intake [69], whereas higher serum leptin levels represent an adequate nutritional status [70]. Leptin is reduced and higher ghrelin levels are observed in subjects with shorter sleep durations [71], as regularly seen in shift workers [58,60,72], leading to a state of increased metabolic stress, which serves as a driver for development of metabolic diseases [73]. In addition, stress, irregular and unhealthy eating patterns and sedentary lifestyle may also lead to disturbances in circadian processes (Figure 1).

### 3.2. Intermittent Fasting as a Lifestyle

Intermittent fasting (IF) has become the center of public attention and, therefore, of more research in recent years [74,75,76]. IF means food deprivation in a daily or hourly time window. Methods carried out on a daily basis are usually linked to the aims of caloric restriction and weight loss. Probably the foremost known forms of day-long fasts are the so-called Leangains and Warrior Diet, in which an 8 h or 4 h eating window is implemented from lunch to dinner or at the end of the day. These fasting forms, also known as time restricted feeding (TRF), are intended to be suitable as a long-term form of nutrition and will therefore be discussed in more detail in the following section.

#### 3.2.1. Intermittent Fasting and Its Influence on Central Clock Genes/Hormones

Recent studies in humans reported that intermittent fasting (IF), especially when feeding time is early or in the middle of the day, results in decreased body weight and fat mass as well as improved blood pressure and insulin sensitivity [77,78,79,80,81]. Due to the time restricted food intake, the body uses less glucose and more lipids and ketones as energy resources [82], resulting in improved glucose and lipid homeostasis [83], preventing mitochondrial ageing processes [84] and deoxyribonucleic acid (DNA) repair by enhancing ketone body levels, indicators for a healthier metabolic phenotype [85]. In addition, a stimulated thermogenic activity in brown adipose tissue leads to a reduction in body fat [86]. Many effects of TRF on either hypothalamic, pituitary, or both, hormones have already been described in literature [18,87,88,89] so its influence on metabolic processes, whether in health or in disease, cannot be neglected.

#### 3.2.2. Intermittent Fasting and Hormonal Pathways

The hypothalamus, part of the endocrine system and nervous system, forms the interface between the nervous and hormonal regulation of metabolism [90,91]. The SCN influences metabolism in peripheral organs by transmitting the time information via circadian CCGs to target organs, but peripheral CGs also show autonomous regulation of metabolic processes and influences CCGs, underlining the bidirectional character of circadian control. The connection between both is described in the review of Mazzoccoli et al., showing that misalignment of the central circadian clock in SCN with unusual light/dark cycles, or alterations in peripheral metabolic processes can both lead to disturbances in circadian rhythmicity [51]. The hormones produced in the hypothalamus can be divided into two different classes—releasing hormones (RH) and inhibiting hormones (IH)—according to their function. RH promotes the release of other hormones like corticotropin releasing hormone (CRH) for secretion of ACTH, whereas IH mitigates their release, like CRH-induced secretion of somatostatin, resulting in suppression of the release of growth hormone (GH) [92,93,94]. After the secretion of RH and IH, they reach the anterior pituitary gland (adenohypophysis) via the portal system, transmitting the hormones to trope cells and stimulating or inhibiting the secretion of somatotropic and glandotropic hormones [95]. They control the hormone release in peripheral glands, like glucocorticoids, influencing cortisol, insulin and glucagon secretion [96]. The two hormones, glucagon and insulin, secreted by the α-(glucagon) and β-(insulin) cells of the pancreas, mainly regulate blood glucose levels [97]. Plasma insulin secretion is increased when blood glucose levels are elevated [98] for stimulating glucose transport to muscle and adipocytes, while gluconeogenesis in the liver is inhibited [99]. In contrast, during hypoglycemia, glucagon secretion is promoted, which increases blood glucose levels by hepatic glucose production [100]. The peak of the circadian insulin secretion is reached in the early morning. Besides this internal rhythm, food intake has a great effect on insulin secretion, resulting in a postprandial increase of insulin and glucose levels [93]. In contrary, TRF reduces fasting insulin levels when implementing in the morning, indicating an enhanced insulin sensitivity [78,101]. Whole-body insulin sensitivity decreases throughout the day [102,103], which is why it seems that food intake at the beginning of the day is preferable, making early TRF the chosen method for improving and preventing metabolic diseases. Insulin and glucagon as the main hormones influencing glucose homoeostasis are regulated by SCN during fasting times but their secretion is sufficiently driven by tissue-specific CCGs during feeding cycles [104]. A change of the eating time from active to inactive phase, can thereby lead to a reset of peripheral clock machinery, leading to shifted phases of peripheral and circadian clocks [105,106]. Organs involved in this complex process are the liver, pancreas, adipose and muscle tissue. The liver has a central role in maintaining glucose homeostasis and is governed by SCN control [107]. However, implementation of TRF has shown that alteration in the fasting/feeding time window results in complete decoupling of the liver clock from the hypothalamic clock. Many of the circadian transcripts found in the liver influence CG *Per2* independently of the hepatocyte clock, highlighting the influence of additional external, hormonal or behavioral clocks [108]. Here, administration of insulin has been shown to induce a phase-advance of PER2. Conversely, a liver-specific *Bmal1* knockout affects glucose homeostasis and *Cry* knockout alters glucagon-induced gluconeogenesis. Both lead to an improvement in glucose tolerance. [109,110]. In muscle, *Rev-Erbα* is the central CG. A deficiency in *Rev-Erbα* results in a deterioration of mitochondrial function [111], implying a deficiency in glucose metabolism, while a knockout of *Bmal1* is associated with impaired muscle glucose uptake and metabolism [112]. Adipocyte-specific *Bmal1* knockout leads to obesity, displaying the increase in food consumption when lacking *Bmal1* [113]. However, in contrast to liver-specific knockout of *Bmal1* [109], no increase in insulin sensitivity could be detected. This finding is supported by results of Basse et al., which demonstrated a different diurnal rhythm of glucose uptake in skeletal muscle and adipose tissue compared to diurnal whole-body insulin tolerance in mice [114]. Metabolic processes in adipose tissue are also driven by other endogenous clocks like adipokines, with adiponectin regulating insulin sensitivity with a peak in the active phase [115]. Adipokines show a circadian rhythmicity but can also be altered with changes in sleep/wake and feeding/fasting cycles, which could explain the increased prevalence of metabolic disorders in shift workers [116]. Furthermore, in pancreatic cells, further autonomous circadian rhythms have been found. Glucose-induced insulin secretion seems to be dependent on the two pancreatic-CGs, *Bmal1* and *Clock*. This was demonstrated in *Clock* and *Bmal1* knockout mice, showing a disturbed glucose tolerance and decrease in insulin secretion [30]. *Clock* and *Bmal1* are also involved in expression of some islet genes and therefore a normal pancreas-specific CG expression is required for sufficient insulin release for glucose homoeostasis [117,118].

During fasting, glucagon stimulates hepatic glucose production. Several studies suggest that glucagon levels also influence the expression of central CGs [119,120]. The effect of restricted feeding on glucagon levels and its influence on CGs was demonstrated by Mukherji et al. They measured a starvation-induced increase in glucagon and free fatty acids (FFA), leading to activation of transcriptional factor cAMP response element-binding protein (CREB) through glucagon and peroxisome proliferator-activated receptor alpha (PPARα) activation through enhanced FFA levels. This results in activated *Per1* and *Per2* expression (glucagon) and an increase in *Rev-Erbα* expression activated by FFA-liganded PPARα [121]. The interplay between peripheral pacemakers and CG expression was also investigated by Jakubowicz et al., who compared the influence of glucagon-like peptide 1, a peptide hormone regulating glucagon and insulin levels [122,123], on CG expression after a single omission of breakfast in healthy individuals compared to individuals with type 2 diabetes. They detected large differences between both groups after breakfast and on fasting days, resulting in the expression of different CGs in healthy compared to type 2 diabetes. In addition, a difference in gene expression was detected between breakfast and fasting in both groups. This shows that metabolism in type 2 diabetes is associated with specific changes in gene expression and breakfast skipping shows metabolic effects in health and disease [124]. The highly complex relationship between feeding/fasting cycles and CGs and CCGs and its positive effect for resetting the circadian clock and preventing chronic metabolic diseases was discussed in more detail in the literature [89,101,125,126,127].

Another metabolism influencing hormone is adiponectin, an adipokine expressed in adipose tissue like leptin, sensitizing the tissue to insulin [128]. Adiponectin level is lower at higher body weights and leads to insulin resistance, a higher fat mass and central fat distribution [129,130,131]. TRF appears to be a valuable method for enhancing adiponectin levels, which was proven by Moro et al. [81]. They randomized 34 resistance-trained males to a regular diet group and to TRF (16 h fasting, 8 h eating). After 8 weeks of intervention they detected a highly significant increase in adiponectin levels whereas leptin levels were lowered, which is in accordance with other literature [132]. In non-obese mice with type 1 diabetes, it was further shown that substitution of insulin with leptin or supplementation of leptin instead of insulin therapy can mimic the effect of insulin monotherapy [133]. Glycated hemoglobin (HbA_1c_), indicator of long-term glycemic control, normalization was achieved with significantly lower glucose fluctuations. Furthermore, a reduction in plasma and tissue lipids was demonstrated, showing the comprehensive effect of leptin on metabolism.

### 3.3. Exercise as a Lifestyle

Within the last decades, physical exercise shifted towards the center of attention due to its multifactorial properties [20,134]. It can be applied in nearly any population of any age-group to improve physiological and psychological parameters and general quality of life [135,136,137,138]. Due to these properties, medicine has started to apply exercise with a personalized approach and moved forward towards an adjuvant therapy in different cohorts [139,140,141]. Besides its features in treating disease, it has also become part of daily life in healthy populations [142,143]. The World Health Organization (WHO) recommends at least 150 min of moderate-intensity, or 75 min of vigorous intensity per week [144]. From this perspective, living a physically active lifestyle is desirable and the urge of physiological improvement to remain healthy and live with a high quality of life has become present through all age groups by an increased application of physical activity trackers, food apps or sleep trackers [145,146,147].

Users of these applications receive plenty of information from their devices; however, it is not entirely clear how nutrition, exercise and sleep interact. Of course, it is well known that a certain number of calories with a specific composition should be consumed daily to perform well in any kind of physical activity. However, dependent on the goal of the individual, a calorie surplus or a reduction of calories is important. Furthermore, composition and meal timing play a major role in physiological performance. Since the management of exercise timing, intensity, duration and frequency and consumption of food in a specific manner, calorie amount and composition of food has not been complex enough, sleep and recovery should also be incorporated. Lack of sleep is the cause of severe psychosocial diseases and also the origin of metabolic diseases since the hormonal cycle is reliant on a regular sleep and wake cycle. Once this cycle is disturbed, our physiological performance decreases rapidly.

As a consequence, it is of interest to shed some light onto a topic that might appear trivial, yet the fragility and complexity of the simple and popular mantra “eat, train, sleep—repeat” might lead us further away from what it is actually supposed to be—a retreat.

#### 3.3.1. Macronutrient Timing

Carbohydrates, mainly glucose, are used in particular by the brain and muscles during physical activity [148,149]. A higher glucose tolerance in the morning [150] seems to make carbohydrate intake more reasonable in the early hours. This has been underpinned by a cross-over study in 10 healthy men that showed an increase in core body temperature (CBT) of up to 8 h and suppression of nocturnal melatonin production following a single carbohydrate-rich evening meal on 3 consecutive days [151], and thus, a shift in the circadian clock. However, eating patterns must be considered in a differentiated way for athletes when a performance limiting factor is the availability of carbohydrates [152]. Moreover, for short recovery times between training sessions (<8 h), recommendations move towards immediate carbohydrate intake after the end of training whereas longer recovery times (>24 h) allow a more flexible intake [153].

Along with carbohydrates, fats represent the main energy reserve in the body. The influence of a nightly high-fat meal was studied in 25 healthy adults [154]. The postprandial response was compared during the day (1:30 p.m.) and at night (1:30 a.m.), with significantly higher circulating triacylglycerol (TAG) levels detected at night. In another trial, there was shown that TAG levels are lower after lunch as compared to breakfast [155], which could be explained by an increased uptake of TAGs into skeletal muscle and brown adipose tissue during the active phase [156]. Burdge et al. concluded that metabolic disease could possibly be prevented if high-fat meals were consumed sooner at lunch.

After their breakdown into amino acids and peptides, proteins are taken up by peptide transporters, mainly peptide transporter 1 (PEPT1), in the intestine [157]. Animal studies suggest that PEPT1 levels exhibit a diurnal rhythmicity, with increased levels at the beginning of the active phase [158]. This implies that protein intake, as well as carbohydrate intake, should occur at the beginning of the day.

#### 3.3.2. Exercise Timing

When it comes to the question of whether exercise should rather take place in the morning or evening, opinions differ tremendously. While some consider their training sessions to be a good way to end the day, others cannot get any rest after a late sports unit. Looking at the normal circadian rhythm, this seems surprising at first. For both strength and endurance training, maximum performance is observed in the afternoon and evening hours [159,160,161]. This is caused by the diurnal rhythm of the core body temperature (CBT). Rhythmicity is controlled by changes in blood flow, and thus, skin temperature of the distal limbs, which reaches its maximum in the late evening and its minimum in the morning [162]. The same processes controlling CBT are responsible for thermoregulation in exercise. This was demonstrated by comparing the initial response of temperature to moderate activity at different times of the day (5:00 a.m., 11:00 a.m., 5:00 p.m. and 11:00 p.m.). Thus, the increase of about 0.75 °C in CBT in the morning is significantly larger than 0.45 °C at the other experimental time points, whereas the increase in skin blood flow measured at the forearm was smaller [163], which is consistent with other literature [164,165]. This indicates that at times of most efficient thermoregulation, the greatest aerobic performance can be observed. For example, in a ramp test performed in 11 cyclists, up to 95% of the maximum power (P_max_), the time to exhaustion (TTE) was determined [166]. Thus, TTE in the evening (6:00 p.m.) was on average 40 s (270 ± 105 s vs. 234 ± 85 s) longer than in the morning (6:00 a.m.). In contrast to 15% increase in power output (±16%), no difference was measured in VO_2mean_ and VO_2peak_. Similar results were obtained in a TTE (increase of 3.66% VO_2max_/s to a maximum load of 95% of VO_2max_) in eight women. This is in agreement with other studies [167,168,169]. However, recent results highlight that large variations in performance can be observed between morning and evening types [170,171,172]. Thomas et al. concluded that exercise in the evening could lead to misalignments in early chronotypes [173].

The results of anaerobic performance and muscle strength tests do not always show consistent results. However, the trend also shows that peak performance tends to be elevated in the afternoon hours [174]. When comparing a force-velocity and a multi-jump test at three different times of the day (9:00 a.m., 2:00 p.m. and 6:00 p.m.; 23 subjects), a 3% increase in performance was detected in the wheel test and 5–7% in the jump test [175]. A further investigation on 19 subjects with extended test times (2:00 a.m., 6:00 a.m., 10:00 a.m., 2:00 p.m., 6:00 p.m., 10:00 p.m.) demonstrated that for P_max_, an increase of 7% at the peak clock time at 17:10 ± 00:52 h was observed. Furthermore, P_peak_ and P_mean_, determined by Wingate test, resulted in an increase of 7.6% and 11.3% and a peak clock time at 5:24 p.m. ± 00:36 h and 6:00 p.m. ± 01:01 h, respectively [176]. This is consistent with other research [177,178,179]. The peak performance in the afternoon hours can also be observed for muscle strength tests [180]. Thus, the research is clear for most types of exercise and indicate that performance is critically influenced by intrinsic factors. This demonstrates that knowledge of chronotype and the general circadian rhythmicity of thermoregulation is an important tool in exercise planning, especially if training sessions are to be performed at maximal load with the lowest possible risk of injury [181].

Besides the influence of normal circadian variations of CBT on exercise performance, the peripheral muscle clock might have a crucial impact on diurnal energy metabolism. Initial results on metabolomic and transcriptomic investigations demonstrated that exercise in the early active phase influences other metabolic processes than exercise in the early rest phase [182]. Adenosine triphosphate (ATP) production and utilization are dependent of hypoxia-inducible factor 1α (HIF1α) production in exercising skeletal muscle under hypoxic conditions. Mason et al. demonstrated that a shift from glycolytic to oxidative metabolic processes can be observed in HIF1α knockout mice, resulting in increased exercise times in concentric and decreased times in eccentric exercise [183]. In this regard, a high HIF1α muscle content seems to be beneficial during exercise with a high content of glycolytic metabolic processes, whereas low levels seem to be advantageous during aerobic exercise. Sato et al. found higher levels of HIF1α only during exercise in the early active phase and a reduction in the levels of transcripts involved in mitochondrial processes, whereas glycolysis-related transcripts were elevated. In addition, they were able to detect increased fat and protein utilization in the early active phase. On the other hand, an increase in energy expenditure was only detected when exercising in the early rest phase. They concluded that energy depletion in skeletal muscle cells is highest in early active hours and thus leads to changes in metabolic processes between the different times of exercise. For this reason, it seems advisable that concentric exercise with high mitochondrial capacity is better performed in the late active phase [182].

#### 3.3.3. Exercise and Other Hormonal Pathways

The effect of exercise on hormonal pathways has often been discussed in literature [184,185,186,187]. A central point of scientific research is the investigation of insulin-independent GLUT4 skeletal muscle expression while exercising [188,189]. GLUT4 is a glucose transporter that regulates glucose uptake in skeletal, cardiac muscles and fat cells, increasing insulin sensitivity and improving glucose metabolism, making exercise an early treatment in insulin resistance and diabetes.

Cortisol is a frequently studied hormone in connection with exercise. Its secretion in adrenal cortex is initiated through HPA axis after release of ACTH [92]. Besides its function as the primary stress hormone [190], cortisol levels also represent the response to metabolic processes by releasing glucocorticoids which increases macronutrient utilization [191]. External factors influencing cortisol levels are meals, hydration and exercise. During exercise, hydration influences the cortisol concentrations, as shown by Maresh et al. Thus, in a dehydrated state (hypohydrated by 5% of body mass), cortisol concentrations were higher than in euhydrated state. Furthermore, an increase in exercise intensity from 70% to 80% VO_2max_ resulted in higher cortisol concentrations before and 20 min after exercise [192]. Cortisol secretion is particularly important during prolonged exercise, as it prevents the esterification of FFAs and stimulates glucose production in the liver [193,194]. Cortisol, which is elevated during chronic stress can lead to the development of disease, from chronic pain to cardiac diseases [190,195]. However, after exercise it falls rapidly and can lead to a decrease in basal cortisol levels during sleep. This was shown in 17 athletes in whom cortisol response to (1) a moderate training, (2) two high-intensity training sessions or (3) no training was measured at night [196]. This phenomenon, termed the exercise-glucocorticoid paradox by Chen et al. 2016 [197], shows that acute exercise induces a stress response, but the long-term effect leads to a reduction in stress hormone production. The cortisol response to exercise at different times of the day (7:00 a.m., 7:00 p.m., 12:00 a.m.) was also studied and it resulted in different cortisol levels (highest cortisol levels at 7:00 a.m., suppression of cortisol release at 12:00 a.m.) [198]. The greatest influence on the increase in cortisol between rest days and exercise days was observed in the measurement at 12:00 a.m., since a lower basal cortisol concentration was determined here, due to the circadian rhythmicity of cortisol secretion (integrated cortisol concentrations 07:00 a.m.: 127.64 ± 6.46 vs. 135.91 ± 12.23; 12:00 a.m.: 74.77 ± 14.29 vs. 103.94 ± 13.36 min·nmol/L on control vs. exercise days). Subsequently, in people with primary chronic insomnia, their condition was found to be associated with increased cortisol secretion and increased HPA axis activity [199]; implementation of high-intensity exercise late in the evening could have a short-term negative effect on sleep quality. This issue has also been investigated by Buman et al. which used surveys to collect data on exercise behavior and sleep quality from 1000 individuals [200]. They distinguished between physical activity >8 h, 4–8 h and <4 h before bedtime and their influence on sleep quality. In their data, no significant difference between the groups was found, which could be associated with a worsening in sleep quality when exercising in the evening. However, the low significance due to the subjectivity of the answers and the lack of physiological measurement data must be viewed critically. Another point that should be considered when discussing exercise and associated cortisol levels, is the adrenal clock of cortisol production. Cortisol concentrations peak in the early morning. This is mediated by adrenal CGs, triggered by light stimuli [93]. Cortisol, besides other glucocorticoids, was found to generate a catabolic status of muscle tissue [201,202]. Therefore, it seems, that especially anaerobic exercise and strength training should be performed in the afternoon, when cortisol levels decrease. This is supported by Bird and Tarpenning, as they showed that the cortisol response of 13 weight-trained men to heavy strength training was significantly lower, when performing at 6:00 p.m. compared to exercise at 6:00 a.m. [203].

**Figure 2:**
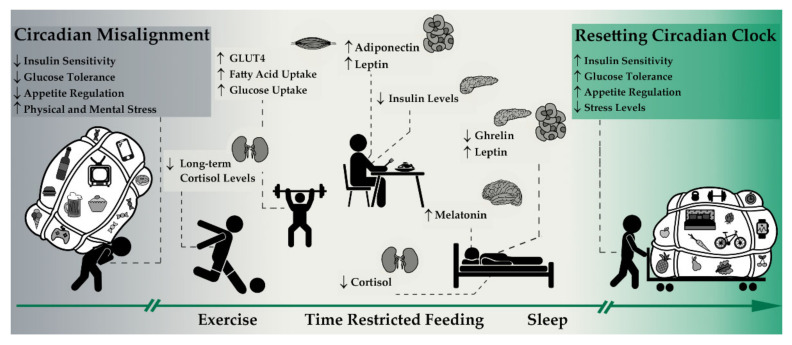
Resetting the circadian clock. Sleep, time restricted feeding (TRF) and exercise affect circadian rhythmicity helping to resynchronize the central with peripheral pacemakers by influencing different metabolic pathways. Exercise particularly affects peripheral processes in muscle and adipose tissues and influences glucose homeostasis. In addition, regular exercise can reduce stress levels by influencing cortisol levels. Besides, glucose homeostasis and hunger/satiety hormones are controlled by TRF. Despite these peripheral influences, sleep also affects central rhythmicity through a direct impact on melatonin and cortisol secretion.

As demonstrated in the previous sections, implementing normal day/night rhythm, exercise and time restricted feeding significantly influence metabolic processes and can thus help to rebalance circadian processes as also shown in Figure 2.

## 4. Discussion

Since the circadian rhythm is already a highly complex system of central and peripherally controlled hormonal and metabolic processes, this system becomes even more complex once TRF and exercise are incorporated [161,204,205]. TRF has been shown to be a tool in regulating glucose and lipid metabolism and increasing insulin sensitivity by directly regulating the blood glucose levels by means of hormones, insulin and glucagon, among others [78,101,119]. This is enhanced by the expression of adiponectin, sensitizing the adipose tissue to insulin and leptin, mimicking the effect of insulin monotherapy [133]. The blood glucose-lowering effect is also observed with the implementation of exercise, here the blood glucose-regulation runs through the peripheral insulin-independent expression of GLUT4 in muscle cells [188,189]. Considering the normal circadian rhythm of macronutrient uptake, with an increased glucose tolerance and a peak activity of PEPT1 for most efficient protein uptake in the morning and with an increased uptake of TAGs in the middle of the day, it seems that TRF with an early feeding phase is the most natural way of feeding, as metabolism is most efficient at this time and the ingested food can thus be best utilized. Some studies also showed more positive effects when feeding time fell in the morning hours compared to late feeding cycles. The late fasting cycle is therefore able to prevent the harmful effects of a nightly high fat diet and the associated increase in TAGs as well as an increased postprandial CBT after an excessive carbohydrate intake and thus a suppression of melatonin expression in the evening. Even though thermoregulatory processes are more efficient in the afternoon and therefore maximal performance is usually maintained at this time, for recreational sports with mostly short durations or moderate intensities no or hardly any negative effect in performance should be noticed by switching exercise time from evening to morning hours. This allows a high flexibility in planning fasting/feeding cycles with exercise, especially for healthy individuals. The situation is different if TRF is used for health purposes like support in the treatment of type 2 diabetes. In this example, exercise should be performed before feeding time, as it results in a significantly higher improvement in blood glucose control [206] and lipid metabolism [207] in contrast to post-meal exercise. However, for other diseases or exercise types, the individual condition of the person must be taken into account. A differentiated metabolic response to morning (fasting) and afternoon resistance exercise was observed in type 1 diabetics, in whom a greater incidence of hyperglycemia events occurred after morning than after afternoon exercise [208]. Summarizing these results, an early feeding time with exercise before feeding seems to result in the greatest improvements in metabolic processes and is preferable for the treatment of diseases, but must always be adjusted to the individual. For athletes, a proper exercise timing as well as an adequate carbohydrate supply during either high-intensity, long duration, or both, exercises seemed to be essential as it could be a limiting factor in maintaining exercise performance [209,210]. Furthermore, replenishing used glycogen immediately after training sessions for better regeneration is important [211]. A slower regeneration is not a problem in recreational exercise with a recommendation of 150 min of moderate-intensity, or 75 min of vigorous intensity per week, if a sufficient amount of carbohydrates is supplied within 24 h following exercise as suggested by Burke et al. However, this should be considered for ambitious recreational athletes as well as competitive athletes with several intensive training sessions per week or day. In that case it should be carefully weighed up whether the eating window should be placed in the midday and evening hours, or—preferably to be applied in the case of an early chronotype—the intensive units should be postponed to the morning hours. Depending on the goal—whether maximum physical performance or health purposes—attention should also be given to a sufficient timeframe for the feeding period. Tinsley et al. showed that a limitation to 4 h of eating per day led to a reduced energy intake. This could be beneficial for health aspects, such as a reduction of body fat in overweight people, but could have a negative effect on athletes’ performances. TRF and exercise have been shown to mainly influence peripheral hormonal processes in specific tissues, such as adipose tissue, pancreas and muscle cells, with less influence on the central control mechanisms [15,18,78,81,89]. In contrast, the normal day/night rhythm may have an enormous influence on the central pacemaker—the SCN—and thus on the melatonin production controlled by the light/dark cycle [212]. Shifts in the circadian melatonin rhythmicity lead to shifts of the central and peripheral pacemaker against each other, which subsequently induces a suppression of the amplitude of the phase, or free running periods of hormone and gene expression [63,213,214]. These disturbances can be considered as the cause of metabolic diseases. Disturbances in the complex circadian system can be observed especially in shift workers since sleep deprivation increases disease risk factors. Furthermore, social jet-lag, in other words, the shift of the social clock against the normal circadian rhythm of the metabolism, as well as the excessive use of light-intensive devices until late in the evening, lead to an increased prevalence of modern diseases [215,216].

## 5. Conclusions

As shown in Figure 2, exercise and TRF are simple options implementable in daily life situations to potentially eliminate symptoms of sleep deprivation and other unhealthy lifestyle behaviors (Figure 1), leading to a disturbed circadian rhythm. However, the potential cause of metabolic disease arising from sleep deprivation may not solely be eliminated by the aforementioned implementation. Therefore, not only symptoms must be treated, but a holistic approach is necessary, which first identifies the disturbing factors on the sleep/wake cycles and successively eliminates them. Adding TRF and exercise is reasonable, and since for most people a significantly higher meal frequency than exercise frequency is achieved and more comprehensive processes can be regulated by TRF compared to exercise, we should probably go from: eat, train, sleep—retreat to sleep, eat, train—retreat.

## Data Availability

Not applicable.
